# The influence of in-groups and out-groups on the theory-of-mind processing: evidence from different ethnic college students

**DOI:** 10.1186/s41235-023-00461-6

**Published:** 2023-01-24

**Authors:** Tingyu Zhu, Lijin Zhang, Ping Wang, Meiqiu Xiang, Xiujuan Wu

**Affiliations:** 1 School of Psychology, Shaanxi Normal University, No. 199, Chang’an South Road, Yanta District, Xi’an, 710062 Shaanxi Province China; 2 Shaanxi Provincial Key Research Center of Child Mental and Behavioral Health, Xi’an, China; 3 Shaanxi Key Laboratory of Behavior and Cognitive Neuroscience, Xi’an, China; 4 School of Educational Science, Liupanshui Normal University, Liupanshui, China

**Keywords:** Theory of mind, Ethnicity, In-group, Out-group, Cross-cultural research

## Abstract

According to previous studies of theory of mind (ToM), social environment and cultural background affect individuals’ cognitive ability to understand other people’s minds. There are cross-group differences in ToM. The present study aimed to examine whether social environment and culture affect the ToM in Uygur and Han groups and whether the individual’s cognitive ToM and affective ToM show in-group advantages. Han and Uygur college students were recruited as participants. The “self/other differentiation task” was used to measure cognitive ToM (Study 1), and the “Yoni task” was used to measure both cognitive and affective ToM (Study 2). We found that Han participants processed the cognitive and affective states of others faster and more accurately than Uygur ones. Uygur and Han participants processed in-group members’ cognitive and affective states faster and more accurately. Furthermore, Uygur participants were more accurate in the cognitive ToM processing of in-group members, while Han participants were faster in the affective ToM processing of in-group members. The findings indicated that ethnic culture and group identify might influence ToM processing. Strengthening exchanges between ethnic groups may enable individuals to better process out-group members’ psychological states.

## Introduction

Theory of mind (ToM) is the individuals’ ability to know and comprehend their own and others’ mental states (wishes, beliefs, intentions, emotions, etc.) and to explain and speculate about the mind and behavior of others (Wellman & Gelman, 1992). The two categories of ToM are cognitive ToM and affective ToM. People’s ability to comprehend their own and others’ beliefs, ideas, or intentions is called cognitive ToM, whereas affective ToM refers to people’s ability to understand their own and others’ feelings (Shamay-Tsoory & Aharon-Peretz, 2007; Shamay-Tsoory et al., 2010).

Since the concept of ToM was proposed, researchers in this field have shown increased interest in understanding whether ToM is an innate, universal ability rarely influenced by social environment factors or a culture-specific ability developed in the context of social interaction (Misailidi & Tsiara, 2021; Mulvey et al., 2016). Some studies have compared the ToM performance of individuals in different countries, regions, ethnicities, and cultural backgrounds (e.g., Dong & Fu, 2007; Lillard, 1998; Wellman et al., 2006), or investigated the influence of social culture on an individual’s ToM by examining differences in individuals’ ToM processing of others in different cultural groups (Gnülta et al., 2020). However, whether ToM processing is universal across cultures (Carruthers, 2013) or influenced by individuals’ cultural experiences (Kobayashi et al., 2006; Navarro & Conway, 2021; Shahaeian et al., 2014) or whether cultural universality and specificity affect children’s ToM development is unclear (Hou et al., 2019). Group identify is also an important factor influencing an individual’s ToM processing (Mulvey et al., 2021). It was found that children who were evaluating the mental states of an in-group member were more accurate than children evaluating the mental states of an out-group member. Children’s group membership significantly affected their ability to consider out-group members’ mental states, even in a minimal group context (Glidden et al., 2021).

There are 56 ethnic groups in China. Although all ethnic groups have the same cultural background, they retain unique cultures. Both Han and Uygur belong to a collectivist culture (He, 2005), but they still show differences in culture. The Han culture is modest, open-minded, and inclusive. The Han people generally use Mandarin in daily life. The Uygur culture is characterized by ecological ethics, openness, pluralism, and regionalism. Uygur people believe in Islam (Hua, 2014). In the context of Chinese culture, the exchanges between various ethnic groups are deepening, and the cultures of various ethnic groups are constantly blending. In addition to using the Uygur language, Uygurs also learn Mandarin. In this unique social environment, we must further explore differences in ToM processing between ethnic groups. Our study focused on whether cultural background and group identify influence adults’ ToM processing.

### Cross-cultural research of ToM development

According to previous studies, children’s ToM develops in a stable and consistent order (Sabbagh et al., 2010; Wellman et al., 2001), which differs for children from different cultural backgrounds (Fang et al., 2009). Specifically, Chinese children first understand "diverse beliefs" and then "knowledge access," while American children do the opposite. Wellman (2006) used the "theory-of-mind scale" to compare Chinese and American children. They found that while both groups of children were passed all tasks at similar ages, the tasks were completed in a different order. For Chinese children, the earliest understanding of cognitive mental states was evident on the Knowledge-ignorance task, whereas for English-speaking children, the earliest understanding was evident on the Diverse-beliefs task. Children in diverse cultural communities receive different information and have diverse experiences of mental states. These differences resulted in different sequences of understanding that are apparent quite early in development. A meta-analysis of the age differences in the Chinese and North American children’s development of understanding false beliefs found that children’s false-belief performance varied across areas by as much as 2 or more years, specifically, Canadian children developed earlier than mainland Chinese and US children whereas Hong Kong Chinese children developed much later. These differences were the product of (and could be used to reveal) the multiple sociocultural and linguistic factors that jointly shape ToM development (Liu et al., 2008). In addition to behavioral studies, Kobayashi (2007) identified changes in brain activation areas between the two groups of 8- and 12-year-old Japanese–English bilingual and English-speaking monolingual children in his fMRI study, showing that the superior temporal gyrus (STG) was primarily activated in American children, whereas the medial prefrontal cortex (mPFC), middle frontal gyrus (MFG), and precuneus were primarily activated in Japanese children, indicating cultural differences in children’s neural activities when they completed the false belief task. They contemplated that the neural correlates of ToM might vary depending on children’s cultural/linguistic backgrounds.

Based on the aforementioned cross-cultural study of children’s ToM, it is clear that overall developmental features underlying children’s ToM are universal, which all begin from scratch and increase with age. At the same time, children’s ToM develops in diverse ways and at varying speeds depending on their culture. In the context of Chinese culture, children of different ethnic groups also perform differently in ToM tasks. Dong & Fu (2008) found Han children scored higher on false belief tasks than Wa and Lahu children. From childhood through adolescence to adulthood, an individual’s ToM capacity is a lifelong process of development and change. With age, the development of ToM becomes more complicated and precise (Dumontheil et al., 2010). To determine whether the specific social and cultural environment influences the individual’s ToM, it is crucial to research the performance of adults’ ToM.

### Adult’s ToM processing and its cross-cultural comparison

Cross-cultural research on adults’ ToM found that they show egocentricity in the ToM processing in collectivist and individualistic cultures and subsequently experience the adjustment phase to overcome egocentricity (Chen & Su, 2011). However, adults in the collectivist culture can overcome the influence of egocentricity earlier and more effectively (Cohen & Gunz, 2002; Luk et al., 2012). Chinese participants representing a collectivist culture were also more accurate in judging others’ social hardship (empathy accuracy) than British participants representing an individualistic culture (Atkins et al., 2016). Wu and Keysar (2007) asked Chinese and American adult pairs to play a communication game that required perspective-taking to evaluate the effect of culture. They found that although members of both cultures could distinguish between their and another person’s perspective, the Chinese used this ability to interpret other people’s actions more effectively due to cultural patterns. Utilizing a cross-cultural perspective, Aival-Naveh et al. (2019) conducted a systematic review of healthy and clinical samples from more than 45 cultures, revealing that mentalizing profiles might vary across cultures (e.g., self-mentalizing is more prominent than other mentalizing in individualistic cultures, self-mentalizing is less prominent than other mentalizing in collectivistic cultures). Besides, linguistic factors, value preferences, and parenting characteristics may explain these differences. However, the above-mentioned cross-cultural studies on adult ToM in various countries all focused on a single component of ToM, either cognitive or affective, without examining the performance of both components simultaneously.

Additionally, Vu et al. (2017) conducted a cross-cultural study on adults in Vietnam and Netherlands using a cartoon picture selection task to investigate the effect of the initiation of individualism and collectivism on the speed and accuracy with which participants infer mental state. Participants in collectivist and individualist priming (with others or alone) were asked to describe an autobiographical situation. The results indicated that Vietnamese participants representing collectivist cultures processed affective ToM faster than cognitive ToM and non-ToM. When individualism was stimulated, Vietnamese participants’ affective ToM process would be less accurate than when collectivism was induced or not induced. On the other hand, individualistic Dutch participants had worse accuracy in processing affective ToM than cognitive ToM and non-ToM. According to these findings, different cultural orientations have varied effects on adults’ cognitive and affective ToM, and the development of adults’ ToM differs depending on their cultural background.

Kobayashi et al. (2006) used the second-order false belief task combined with functional magnetic resonance imaging (fMRI) to examine the cognitive ToM of 16 native English-speaking American adults and 16 bilingual (English, Japanese) Japanese adults. They found that the ventromedial right inferior frontal gyrus (IFG) was more important for processing ToM specific to the Japanese language, and the left IFG and left insular cortex were more important for processing ToM in English. It demonstrated that culture and language might affect the processing of ToM and IFG.

Navarro and Conway (2021) tested bilingual adults and monolingual adults on the director task to reveal the linguistic and cultural effect on ToM. They found that bilingual adults outperformed monolinguals in perspective-dependent trials of the director task but not control trials. Rubio-Fernandez and Glucksberg (2012) also found that bilingualism affected adults’ abilities to reason about other people’s beliefs. Specifically, they used a traditional false-belief task coupled with an eye-tracking technique and found that bilinguals were less susceptible to this egocentric bias than monolinguals. These research studies suggested that bilingualism was associated with an individual’s ability to account for another person’s perspective. In China, both Han and Uygur learn English, Uygur also use their own Uygur language, so they may be bilingual or multilingual. This may also affect their ToM processing.

Although many studies have illustrated cross-cultural differences in ToM processing, some studies comparing Western (individualist) and Chinese (collectivist) societies have not found cross-cultural differences. For example, Wang et al. (2019) used two perspective-taking tasks to test British (independent) and Taiwanese (interdependent) participants. The results revealed similar alter-centric and egocentric interferences across the two cultural groups, and the shared biases indicated similarities rather than differences in perspective-taking across cultures. Bradford et al. (2018) also found the core and potentially universal similarities in the ToM mechanism across Western and Chinese cultures. They used a false-belief task to test Western and Chinese adults and found that participants from both cultures were slower to shift from Self-to-Other than from Other-to-Self. Despite differences in collectivism scores, culture did not influence task performance, with similar results found for Western and non-Western participants.

### Differences in ToM processing between in-group and out-group

Further, research suggests that individuals’ mental-state reasoning abilities may be more accurate when evaluating in-group members than out-group members (Gönültaş et al., 2020). It may be that children and adolescents have more difficulty imagining the mental state of out-group members than in-group members (Mulvey et al., 2021). Gönültaş et al. (2020) examined Turkish children’s (*M*_age_ = 11.66 years) mindreading and general reasoning about in-group members (Turks), similar out-group members (Syrians within Turkey), and dissimilar out-group members (Northern Europeans). They found that whereas children’s general reasoning about three groups was equivalent, the accuracy of mental state inferences differed by target, with more accurate mindreading of in-group targets compared to both sets of out-group targets.

Elfenbein and Ambady (2003) found that individuals would also display in-group advantages when processing the psychological states of others due to modest differences in nonverbal and emotional information between various races or national cultures. Specifically, for Chinese in China and the USA, Chinese Americans, and non-Asian Americans, the accuracy and speed of judging Chinese and American emotions were greater, with greater participant exposure to the group displaying the expressions. Likewise, Tibetans in China and Africans in the USA were faster and more accurate when judging emotions expressed by host versus non-host society members. They thought the universal affect system governing emotional expression might be characterized by subtle differences in style across cultures. Ferguson et al. (2018) used the avatar visual perspective-taking task to examine whether the age of an observed person (adult vs. child avatar) influences adults’ visual perspective-taking. They found that alter-centric interference was reduced or eliminated when a child avatar was present, suggesting that adults did not automatically decode a child avatar’s perspective. The findings argued against a purely attentional basis for the alter-centric effect and suggested that mentalizing and directional processes modulated automatic visual perspective-taking that was strongly influenced by experimental context.

Adams (2010) investigated the affective ToM of native English-speaking American and Japanese bilingual (Japanese, English) adults using the "reading the mind with eyes" task and brain imaging technology and their understanding of other people’s affective states. The findings revealed that participants speculated more accurately about the affective state of their ethnic group rather than other ethnic groups. Furthermore, the posterior superior temporal sulcus (PSTS), involved in detecting and responding to cultural cues conveyed by the eyes on both sides of the brain, and the inferior frontal gyrus (IFG), influenced by specific cultural patterns, were both significantly affected.

### The present study

China is a multi-ethnic country where Han and national minorities with distinct subcultural social milieus live together, interact, and assimilate the Chinese culture. As a result, this research posed the main aim: Will the fact that someone is from a different culture influence ToM performance when thinking of someone from the other group? We also want to explore Will the ToM work differently across ethnic groups as a result of their social surroundings and their own culture. This study examined differences between ethnic adults in ToM performance and the consistency of group identity’s influence on cognitive and affective ToM. We recruited Uygur and Han college students as the participants because of apparent differences in names and facial features between the two groups, helping the participants differentiate between the two ethnic groups in the ToM tasks. Therefore, this comparison allowed us to explore the influence of social–environmental factors on an individual’s ToM.

In our study, two experiments were conducted to explore the performance of Uygur and Han college students in ToM processing and the performance of in-group and out-group members in cognitive ToM processing and affective ToM processing. Based on previous research studies, ToM should not be considered a monolithic construct, and should instead be explored and measured as multiple domains (Navarro, 2022). There were differences in ToM tasks applicable to adults and children (Warnell & Redcay, 2019). And the cognitive and affective ToM had different constructs (Raimo et al., 2022). The Self/Other Differentiation task and Yoni task were often used by adults (Bradford et al., 2015; Shamay-Tsoory & Aharon-Peretz, 2007) and were well used in the context of Chinese culture (Ge, 2022). So, we chose these two tasks in our study.

Study 1 included the computerized classic false belief task measuring individual cognitive ToM—Self/Other Differentiation Task. It was a 2 (ethnicity: Uygur, Han) × 2 (group: in-group, out-group) × 2 (task conditions: expected, unexpected) mixed experiment design in which ethnicity was the between-subject variable and group and task conditions were the within-subject variables. In Study 2, the Yoni task, which comprehensively measures individual cognitive ToM and affective ToM, was used to examine the differences between Uygur and Han adults’ cognitive ToM and affective ToM, as well as the influence of group identification on cognitive ToM processing and affective ToM processing. It was a 2 (ethnicity: Uygur, Han) × 2 (group: in-group, out-group) × 2 (task conditions: cognitive, affective) mixed experiment design in which ethnicity was the between-subject variable and group and task conditions were the within-subject variables.

## Study 1

### Methods

#### Participants

Participants were 114 college students from two colleges in Xi’an, Shaanxi Province, with 57 Uygur and 57 Han ethnicities in each group (18–23 years old). The two groups were matched in gender and age. Eight participants were excluded from each ethnicity. The sample size of this investigation was 54 at least, as assessed by G*Power software (*α* = 0.05, power = 0.95, effect size *f* = 0.25) (Faul et al., 2007), the number of participants in this study meets the requirements. Twenty-three Uygur were "Min kao Han" students;^1^ they were able to communicate, write and read fluently in Chinese. These students had got Chinese language instruction before enrolling in university, and their courses were identical to those taken by local Han students. The other 26 were bilingual students, who had been learning Chinese since kindergarten or primary school. They had all completed two years of pre-university schooling and were fluent in Chinese before beginning formal undergraduate education. All participants’ visual acuity or adjusted visual acuity was normal, without color blindness or color weakness. After the experiment, the participants were given volunteer service certificates and small gifts. The ethical Committee for Scientific Research at the corresponding author’s institution approved all materials and procedures. This study gained the consent of the participants themselves and signed an informed consent form (Table 1).

**Table 1:** Participants demographics and matching

	Female	Male	*M*_age_ ± SD_age_ (years)
Uygur	27 (26)	30 (23)	20.84 ± 1.07
Han	27 (26)	30 (23)	20.56 ± 1.35

The number in the bracket was the number after exclusion. Participants whose reaction time or error rate above the average of this group by 3 standard deviations were excluded. Correspondingly, participants who matched gender and age were excluded from the other group. Table 3 is the same

#### Materials

(1) Questionnaire of typical names of Uygur and Han male and female students.

We used the questionnaire to pick out Uygur and Han’s typical names. Because the names of Han and Uygur are very different, these typical names were used to distinguish the in-group and out-group of Han and Uygur in the subsequent experiments.

There were 12 typical male and female names (each with 3 characters) of Uygur and Han ethnicities (Uygur males such as "买买提" (Maimaiti) and females such as "古丽仙" (GU Lixian); Han males such as "王志强" (WANG Zhiqiang), females such as "李文静" (LI Wenjing)). The tasks of male participants in this study were all male names to avoid the influence of gender characteristics. The names of the tasks for the females were all females’ names, and the followings were the same.

(2) Pictures of Uygur and Han male and female adults.

A total of four adult photographs were selected from the visual China website (https://www.vcg.com/), one male and one female of Uygur and Han ethnicity. All Uygurs wear their national costumes.

(3) Self/other differentiation Tasks.

**Fig. 1:**
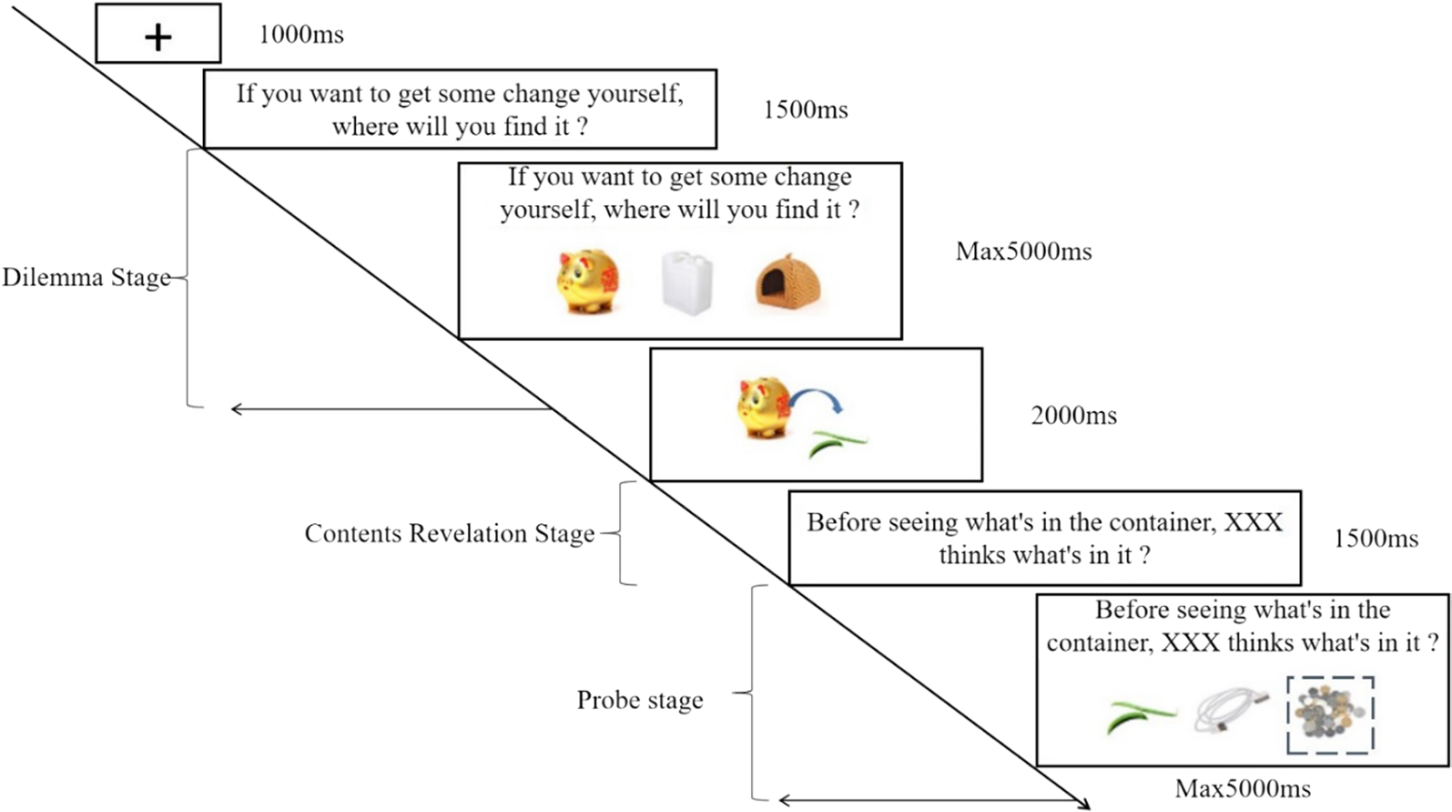
Flowchart of single trial under unexpected conditions of Self/Other Differentiation Task. *Note*. Dilemma stage (self, others: Uygur, Han), contents revelation stage (expected, unexpected), probe stage (self, others: Uygur, Han). In our experiment, English in the original task was translated into Chinese, the followings were the same. For the convenience of reading, we have dotted the correct answers

The self/other differentiation task was an E-prime procedural task designed by Bradford et al. (2015) for adult’s ToM measures based on the classic unexpected content task (Perner et al., 1987). In our experiment, English in the original task was translated into Chinese. There were 80 trials of Self/Other Differentiation Tasks, including 8 practice trials and 72 formal trials. The fixation point “+” of 1000 ms was presented at the beginning of each trial. Then, it entered into three task stages: dilemma stage, contents revelation stage, and probe stage in turn (see Fig. 1 for specific flow). Practice tasks followed the same format as formal tasks, with the exception that in formal activities, participants needed to infer the beliefs of themselves/others.

*Dilemma stage*: Determine the state of belief. Participants were asked to choose a container containing specific items from three container pictures. The stage was divided into self-pointing ("Where will you find it?") and pointing to others ("Where will he/she find it?"), the names pointed out by others were typical names of Uygur and Han ethnicities, and they matched the gender of the participants. In the practice task, the participants were only asked to select certain specific items (for example, "Please select the red schoolbag"). In the formal experiment, within the limited reaction time of 5000 ms, if the participant did not respond or made a wrong response, a red “×” prompt would appear for 1000 ms, and the subsequent experiment would continue. Ascertain that the participants had established the belief that something must be in a specific container.

*Contents revelation stage*: After selecting the correct container for a specific item in the dilemma stage, the participant entered the contents revelation stage, presenting the selected container and the actual items in the container. Items may be expected (for example, the piggy bank contains coins) or unexpected (for example, the piggy bank contains beans); the trials for expected and unexpected items were split into half (in practice tasks, they were all expected items).

*Probe stage*: This was the core stage to evaluate an individual’s ability to infer self/others’ beliefs. Before seeing the contents of the container, the participants were asked to assess their own belief state (self-pointing) or that of others (others pointing). The sentence pattern and length of the probing question were matched under the conditions of self and others, for example, "Before seeing the contents in the container, you/Wang Zhiqiang think what’s in it? " or "If you haven’t seen the contents of the container, you/Wang Zhiqiang think what’s in it?". See Table 2 for the number of trials under different task conditions in Self/Other Differentiation Tasks.

**Table 2:** Number of trials under different task conditions in self/other differentiation task

	Dilemma stage (self)			Dilemma stage (others)			Total trials
	Probe stage (self)	Probe stage (others: Uygur, Han)	Distracters	Probe stage (self)	Probe stage (others: Uygur, Han)	Distracters	
Contents revelation stage: expected	4	8 (4 + 4)	8	4	4 (2 + 2)	8	36
Contents revelation stage: unexpected	4	8 (4 + 4)	8	4	4 (2 + 2)	8	36
Total trials	8	16	16	8	8	16	72

**Table 3:** Participants demographics and matching

	Female	Male	*M*_age_ ± *SD*_age_ (years)
Uygur	25	28 (27)	20.59 ± 1.13
Han	25	28 (27)	20.91 ± 1.09

In order to understand whether Uygur and Han people had differences in the processing of the cognitive psychological state of in-group and out-group members, the reaction time and error rate of the dilemma stage pointing to self and the probe stage pointing to others were analyzed. The setting of self-directed trials in the dilemma stage and probe stage and others’ trials in the dilemma stage was to prevent the participants from expecting answers.

Participants need to pick one of the three alternative pictures in the same row (as shown in Fig. 1) as the answer. The test was conducted on the computer, and the number labels of ‘1’, ‘2’ and ‘3’ were attached to the keys of ‘F’, ‘G’ and ‘H’ in the center of the keyboard, and the three alternative pictures in each question corresponded to ‘1’, ‘2’ and ‘3’ from left to right. Participant pressed the button corresponding to the selected picture, then proceeded to the next trial. Interference items were introduced in the probe stage to eliminate participants’ expectations of the correct response and to ensure that the key selection was done after reading the questions (for example, "What color is the pencil case?"). Half of the interference items appeared after expected items, and half of them appeared after unexpected items. In the practice trials, participants were asked to choose what was in the container just presented (true belief). All the color pictures were from the Internet and were presented in the center.

of the white screen, and the question text was 18 black song typeface. Finally, the reaction time and error rate were recorded.

#### Procedure

First, a questionnaire on typical names of Uygur and Han male/female students was distributed, and the participants were asked to choose the most typical names of Uygur and Han ethnicity. Then, the participants were shown photographs of Uygur and Han people of the same sex. After correctly identifying the two ethnicities in the pictures, informing the participants that the names of the characters in the Uygur and Han pictures were the typical names chosen previously, and compiling the typical names of all ethnic groups chosen by the participants into the Self/Other Differentiation Task, the participants were required to complete the Self/Other Differentiation Task. Before the task starts, the experimenter informed the rules and explained the demonstration. After that, the participants conducted practice trials. After the participants understood the specific process of the experiment, they would be formally tested. The entire procedure took approximately 20–30 min, the subjects would have four breaks to have rest.

## Results

**Fig. 2:**
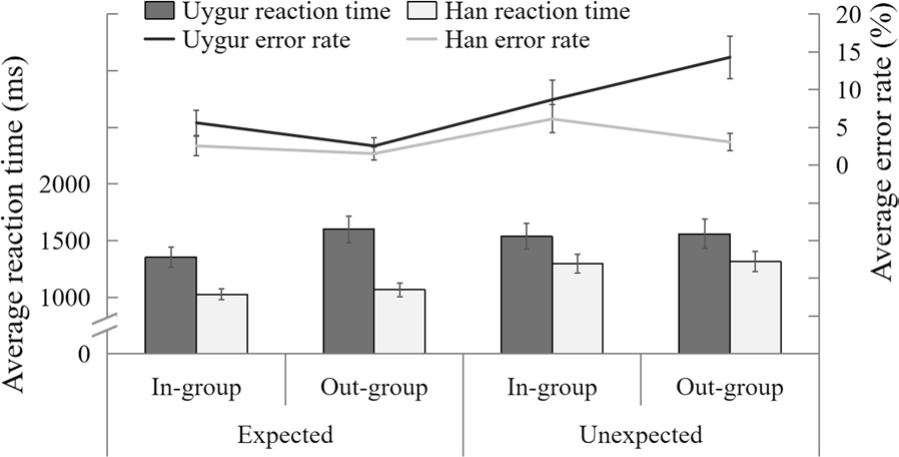
Average reaction time and the error rate of Uygur and Han Ethnicity under different task conditions in self/other differentiation task

To explore whether there were differences in the processing of cognitive mental state of others from in-group and out-group between Uygur and Han people, we used 2 (ethnicities: Uygur and Han people) × 2 (group identification: in-group and out-group) × 2 (task conditions: expected, unexpected) repeated measurement variance analysis for the reaction time (correct reaction time) and the error rate of Self/Other Differentiation Task (see Fig. 2).

The analysis of variance of reaction time showed that the main effect of ethnicity was significant, *F*(1, 96) = 9.49, *p* = 0.007, ${\eta }_{p}^{2}$ = 0.09. The reaction time of Han ethnicity was significantly shorter than that of Uygur ethnicity. The main effect of group identify was significant, *F*(1, 96) = 4.05, *p* = 0.035, ${\eta }_{p}^{2}$ = 0.04. The reaction time of the participants to the in-group was significantly shorter than that to the out-group. The main effect of ToM condition was significant, *F*(1, 96) = 9.73, *p* = 0.003, ${\eta }_{p}^{2}$ = 0.09, the reaction time of participants under expected conditions was significantly shorter than that under unexpected conditions. The interaction was not significant.

The result of variance analysis of the error rate showed that the main effect of ethnicity was significant, *F*(1, 96) = 8.26, *p* = 0.006, ${\eta }_{p}^{2}$ = 0.08. The error rate of Han ethnicity was significantly lower than that of Uygur ethnicity. The main effect of task conditions was significant, *F*(1, 96) = 12.87, *p* = 0.005, ${\eta }_{p}^{2}$ = 0.12. The error rate of participants under expected conditions was significantly lower than that under unexpected conditions. The three-way interaction among ethnicities, group identification and task conditions was significant, *F*(1, 96) = 6.28, *p* = 0.004, ${\eta }_{p}^{2}$ = 0.06. Simple effect test showed that under unexpected conditions, the interaction between ethnicities and group identification was significant, *F*(1, 96) = 7.11, *p* < 0.01, ${\eta }_{p}^{2}$ = 0.07. Simple effect test showed that the error rate of Uygur people’s reaction to the in-group was significantly lower than that of the out-group (*p* = 0.033), and there was no significant difference between the error rate of Han’s reaction to the in-group and the out-group. Under expected conditions, the interaction between ethnic groups was not significant, *F*(1, 96) = 0.62, *p* > 0.05. Other main effects and interactions were not significant.

According to the findings, there were significant differences in cognitive ToM between Uygur and Han participants, with Han participants processing cognitive states faster and more accurately than Uygur participants. The processing of other people’s cognitive states by Uygur and Han people was influenced by group factors, and processing other people’s cognitive states in in-group was faster. However, only Uygur demonstrate an in-group advantage in cognitive ToM processing in the error rate, with no difference between the in-group and out-group of Han participants. In order to further examine whether the influence of group factors on cognitive ToM and affective ToM processing was consistent, study 2 would further examine the inference of cognitive state and affective state of others in the in- and out-groups of Uygur and Han participants, and the influence of group factors on the two groups of participants in the processing of two kinds of ToM.

## Discussion

In this study, cognitive ToM processing differed in the unexpected false belief condition and the expected non-false belief condition. Consistent with the previous findings (Bradford et al., 2015), our results also showed that the reaction time was longer in the false belief condition than in the non-false belief condition, and the error rate in the false belief condition was higher than in the genuine belief condition. In the false belief condition, conflict occurred when one’s belief states were inconsistent with those of others. Individuals must use greater cognitive resources to process the psychological states of others, resulting in longer reaction times and higher error rates. In the case of non-false belief, on the other hand, one’s and others’ belief states were consistent without conflict. Individuals had a faster reaction time and a reduced error rate. In the false belief task, this difference represented the basic substance of cognitive ToM processing, exhibiting cross-cultural consistency and similarity across diverse cultural backgrounds.

Notably, we found that only Uygur participants showed an in-group advantage in the error rate of cognitive ToM tasks. In the false belief task, Uygur participants could estimate the cognitive state of in-group members more accurately, but the difference between Han in-group and out-group members in processing the cognitive psychological states of others was insignificant. This may be because Han participants were not familiar with Uygur names and need more time to process, so they only showed in-group advantage in reaction time, but not in error rate. The core of the self/other differentiation task is to distinguish whether one’s and others’ belief states are consistent (Wang et al., 2008). Han participants may have less impulsive qualities than Uygur participants. They can better restrict their belief states and accurately discriminate between their own and others’ belief states when the belief states are different. Individuals may accurately infer their psychological states and discern whether they are from the in-group or the out-group as long as they can distinguish between their own and others’ belief states. Bradford et al. (2018) used the Self/Other Differentiation task to test Chinese and Western participants, and they did not find cross-cultural difference. What is different from our study is that they used the Chinese version for Chinese participants and English version for Western participants. So, in our research, language may have an impact on Uygur’s ToM processing.

Although the result of reaction time showed that Uyghur and Han participants will have an in-group advantage in cognitive ToM processing, the result of error rate only showed in-group advantage in Uyghur participants which was inconsistent with the result that Adams et al. (2010) found that Japanese adults and American adults both had more accurate affective inference about in-group members. In their research, it was found that American adults and Japanese adults were more accurate in inferring the affective state of in-group members. In order to further verify the influence of in- and out-groups on the processing of cognitive ToM, and whether the influence of group identify on the cognitive ToM processing and affective ToM processing was consistent, the Yoni task, which comprehensively measures individual cognitive and affective ToM, was used in Study 2 to examine the differences between cognitive and affective ToM of Uygur and Han adults, and the influence of group identify on individual’s ToM processing in different ToM tasks.

## Study 2

In study 2, we wanted to further explore whether there were differences in the affective ToM processing among participants of different ethnicities. Yoni task was used to examine whether the cognitive ToM and affective ToM of Uygur and Han adults would show differences due to the influence of social culture and whether individual’s cognitive ToM processing and affective ToM processing would show in-group advantages.

### Methods

#### Participants

Another 106 college students from the same university as study 1 were recruited as participants, 53 from Uygur and 53 from Han (18–23 years old). The two groups were matched in gender and age. Among the valid Uygur samples, 25 were "Min kao Han" and 27 were "bilingual" students. All participants’ vision or corrected vision was normal, without color blindness or color weakness. After the experiment, volunteers would get volunteer service certificates and small gifts. The Ethical Committee for Scientific Research at the corresponding author’s institution approved all materials and procedures. This study gained the consent of the participants themselves and signed an informed consent form.

#### Materials

(1) Typical names of Uygur and Han male and female students.

According to the investigation results of typical names of Uygur and Han male and female students in study 1, the same typical names of Uygur and Han male and female students were selected as the name of the target person in the follow-up experiment.

(2) Uygur and Han male and female face pictures.

Five male and 5 female adults of Uygur and Han ethnicity who did not participate in the experiment were selected to take face pictures of five different eye gaze directions (head up, upper left, upper right, lower left and lower right) under three emotions (neutral, positive and negative). Each person took 15 face pictures, and a total of 300 pictures were taken. Then, four graduate students majoring in psychology evaluated the ethnicity, emotional state and eye gaze direction of all the pictures, eliminated 43 pictures with inconsistent opinions (Uygur: 4 for males and 10 for females; Han: 14 for males and 15 for females), and left 257 pictures. Then 60 college students who did not participate in face picture shooting and Experiment 2 (15 men and women of Uygur and Han ethnicity) were asked to judge the ethnicity, emotional state and eye gaze direction of their own face pictures of the same gender, and 46 pictures with significant ethnic differences in reaction time and error rate in judging their own face pictures were excluded (Uygur: 11 males, 17 females; Han: 8 males, 10 females). The remaining pictures of Uygur and Han ethnicity were matched one-to-one in emotional state and eye gaze direction, and 23 mismatched pictures were eliminated (Uygur: 14 for males; Han: 7 for males and 2 for females). Finally, a total of 188 face pictures (46 pictures for Uygur males, 46 pictures for Han males, 48 pictures for Uygur females and 48 pictures for Han females) were used in the following task.

(3) Yoni task.

**Fig. 3:**
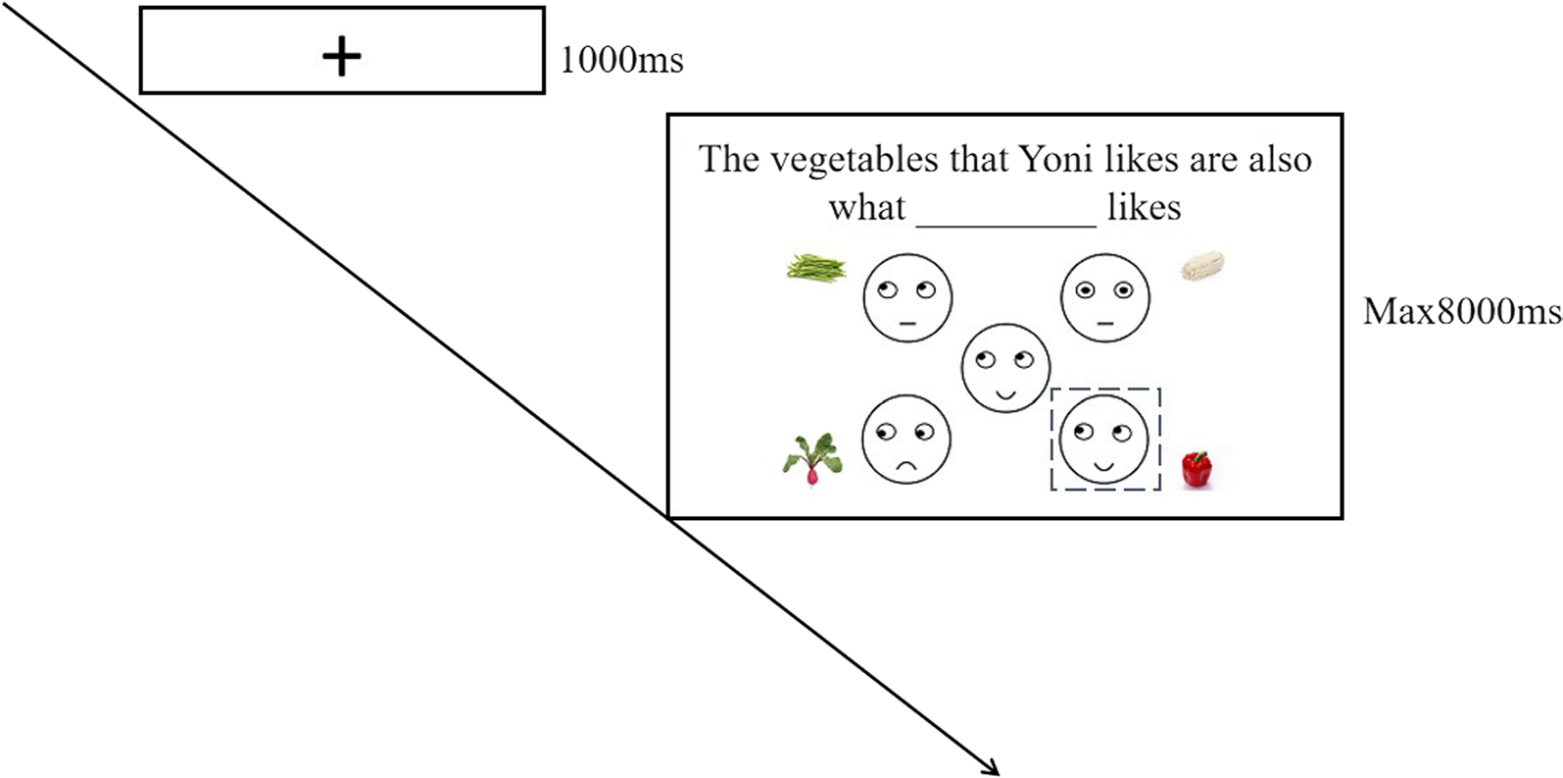
Flowchart of a single trial of Yoni task. *Note*. For the convenience of reading, we have dotted the correct answers

Shamay-Tsoory et al. (2007) created the Yoni task based on the Charlie task (the task of evaluating an individual’s ToM ability based on the inference of linguistic cues and eye gaze signals) defined by Baron-Cohen (1994), which was an E-Prime program task for measuring adult’s ToM. The goal of this task was to deduce the target person’s psychological states using language and facial indicators (such as eye gaze direction and facial expression) (see Fig. 3 for specific experimental flow).

**Fig. 4:**
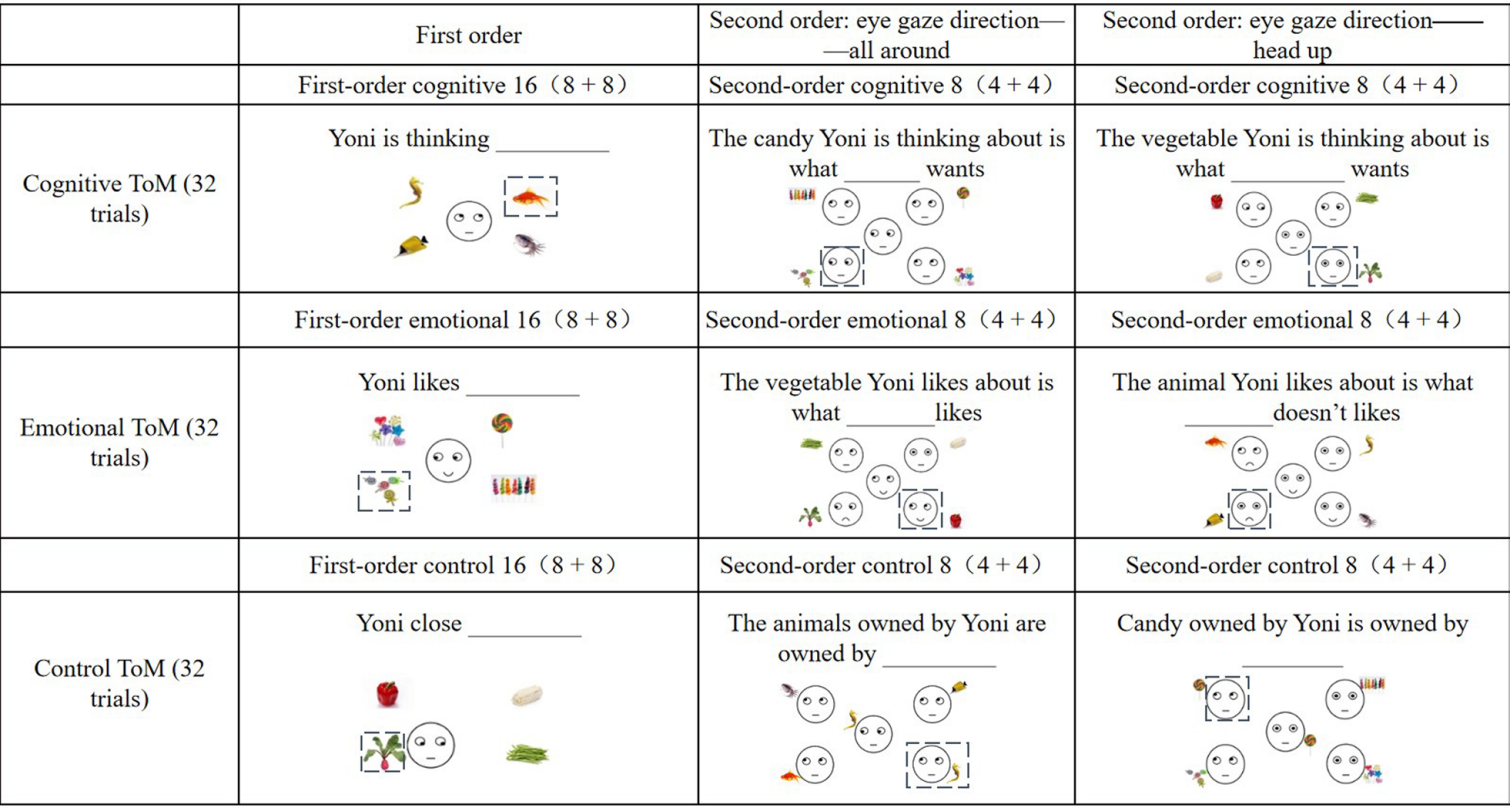
Trials and examples of different task conditions in the Yoni task. *Note.* Only an example of the exercise task is shown in the figure, in the formal experiment, the face pictures of cartoon characters were replaced with Uygur and Han face pictures of the same gender as the subjects. For the convenience of reading, we have dotted the correct answers

Yoni task included practice experiments and formal experiments. The process of the two kinds of experiments was the same, with the exception that in practice experiments, actual people’s faces were replaced with cartoon characters’ faces (Yoni). Three conditions were included in the Yoni tasks: cognitive ToM conditions, affective ToM conditions, and control conditions (In each condition, half of the trials were Uygur faces, and the other half were Han faces). The control condition, for example, required participants to carefully read the task requirements in order to avoid people focusing just on the eye gaze direction of the characters in the picture and neglecting the verbal indications in order to choose the correct response. Furthermore, both first-order and second-order activities were included in these three types of tasks. The target character’s facial and linguistic cues were neutral in the cognitive condition and supply no affective information; in the affective condition, these hints provided affective information. In the first-order task, four pictures of alternative items were presented in four corners of the screen. The participants needed to choose the most appropriate one from the four alternative pictures according to the text clues and the facial clues of the target person (for example, the linguistic clues under the condition of the first-order cognitive ToM are: LI wenjing is thinking ____; The linguistic clue under the condition of the first-order affective ToM is: LI wenjing likes ____). In the second-order task, four alternative face pictures were presented in the four corners of the screen, participants needed to understand the relationship between the psychological state of each person in the four alternative pictures and the psychological state of the central target figure to choose the correct answer (for example, the linguistic clue under the second-order cognitive ToM is: the candy that LI wenjing is thinking about is what ____ wants; The linguistic clues under the second-order affective ToM are: the fruit LI wenjing likes is what ____ likes or the fruit LI wenjing likes is what ____ doesn’t like) (see Fig. 4).

Shamay-Tsoory and Aharon-Peretz (2007) found that some participants neglected language cues and made choices directly according to the direction of the target’s eye gaze. However, when the target person’s eye gaze direction was head-up, the participants must choose according to language cues and facial cues. Therefore, the eye gaze direction of the target person’s head-up was also used as a control condition (see Fig. 4).

The test was conducted on a computer. The words "upper left, upper right, lower left and lower right" were labeled on the "E, U, X and M" on the keyboard, respectively. In each test, the gaze point “+” of 1000 ms would be presented initially at the start, followed by a facial picture of the target individual in the middle of the screen. Four colorful pictures (upper left, upper right, lower left and lower right) would appear in the four corners of the screen (upper left, upper right, lower left, and lower right). In the upper left corner of the picture, there would be a question about the target person, participants should evaluate and choose which picture in the four corners of the screen was the best one to fill in the blank space of the question according to these text clues and the information of the target person’s facial clues (such as eye gaze direction and facial expression). The participant pressed the button corresponding to the selected picture and then entered the next trial. All the color pictures were from the Internet and presented on the white screen, and the words of language clues were all on the 18th black song typeface. Finally, the reaction time and error rate of the participants were recorded. The gender of the photograph matched the gender of the participant to avoid the possible influence of gender issues. Participants with an error rate of more than 50% percent under controlled conditions would be eliminated.

#### Procedure

Before beginning the task, the experimenter described the Yoni task’s rules and demonstrated the task, which was followed by practice experiments. After the participants understood the specific process of the experiment, they would see the pictures of the same gender target figures of Uygur and Han ethnicities in the Yoni task, respectively. After the participants accurately named the two ethnicities in the picture, told them the names of the characters in the picture, and finally executed a formal experiment. The whole process took about 15–20 min, the participants would have three breaks to have rest.

## Results

**Fig. 5:**
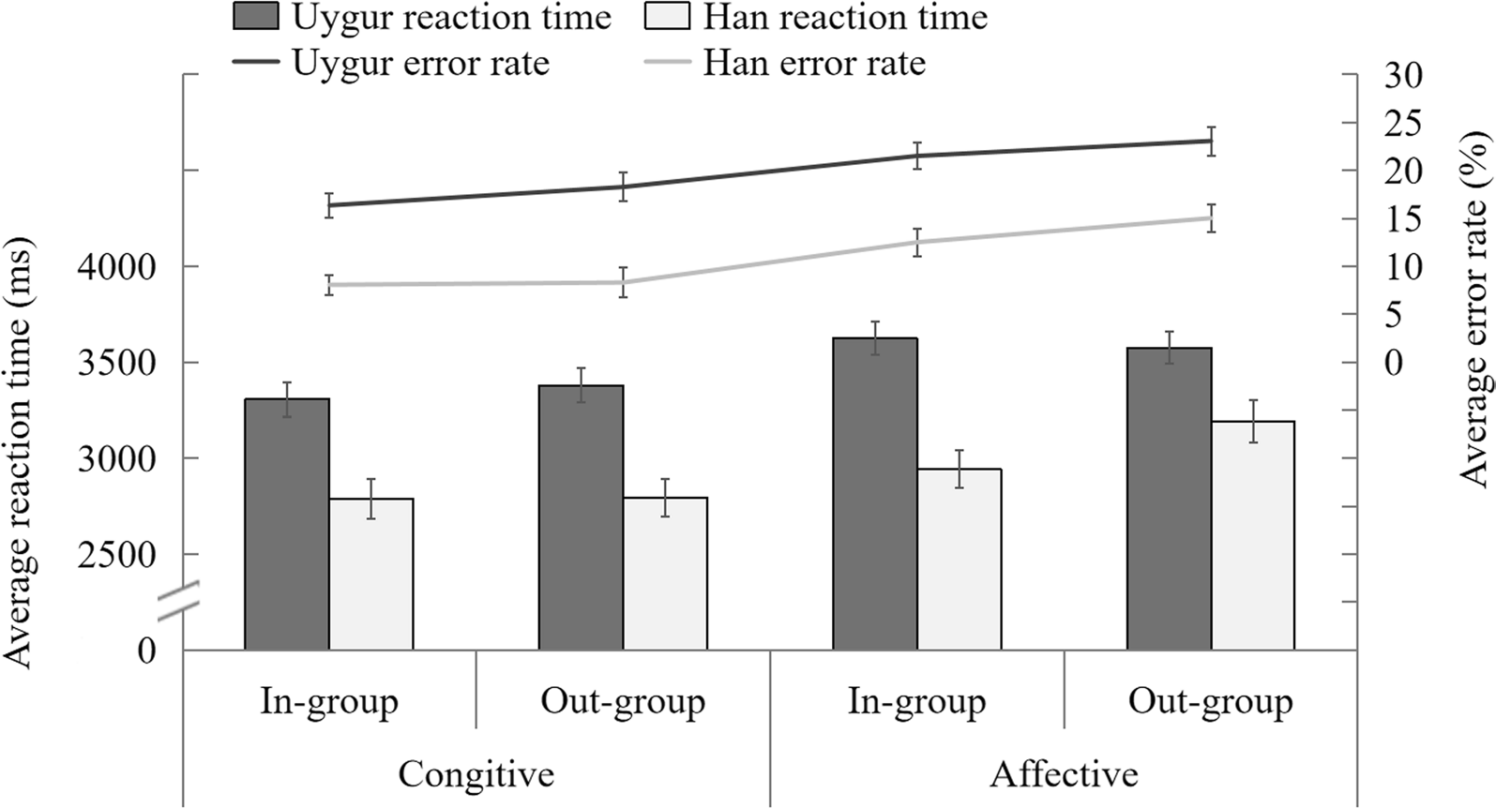
Average reaction time and the error rate of Uygur and Han Ethnicity under different conditions in Yoni task

To find out whether there were differences in the processing (reaction time and error rate) of other people’s psychological state between Uygur and Han ethnicities under cognitive and affective conditions, we used 2 (ethnicities: Uygur and Han ethnicities) × 2 (group identify: in-group and out-group) × 2 (task conditions: cognitive, affective) repeated measurement variance analysis for the reaction time (correct reaction test times) and error rate in the Yoni task (see Fig. 5).

The analysis of variance at reaction time showed that the main effect of ethnicity was significant, *F*(1, 102) = 20.455, *p* < 0.001, ${\eta }_{p}^{2}$ = 0.167. The reaction time of Han ethnicity was significantly shorter than that of Uygur ethnicity. The main effect of group identification was significant, *F*(1, 102) = 5.260, *p* = 0.035, ${\eta }_{p}^{2}$ = 0.049. The reaction time of participants to the in-group was significantly shorter than that of the out-group. The main effect of the task was significant, *F*(1, 102) = 38.674, *p* < 0.001, ${\eta }_{p}^{2}$ = 0.275. The reaction time of participants in cognitive ToM tasks was significantly shorter than that in affective ToM tasks. The interaction among ethnicities, groups and task conditions was significant, *F*(1, 102) = 9.730, *p* = 0.007, ${\eta }_{p}^{2}$ = 0.087. Simple effect test indicated that under the condition of affective ToM, the interaction between ethnicities and groups was significant, *F*(1, 102) = 11.509, *p* = 0.08, ${\eta }_{p}^{2}$ = 0.101. A simple effect test showed that the reaction time of the Han ethnicity to the in-group was significantly shorter than that of the out-group, *p* < 0.001, and there was no significant difference between the reaction time of the Uygur ethnicity to the in-group and the out-group. Under the condition of cognitive ToM, the interaction between ethnicities and groups was not significant, *F*(1, 102) = 0.662, *p* = 0.132, ${\eta }_{p}^{2}$ = 0.006. Other interactions were not significant.

The result of variance analysis of the error rate showed that the main effect of ethnicity was significant, *F*(1, 102) = 33.86, *p* < 0.001, ${\eta }_{p}^{2}$ = 0.25. The error rate of Han ethnicity was significantly lower than that of Uygur ethnicity. The main effect of group identification was significant, *F*(1, 102) = 7.36, *p* = 0.006, ${\eta }_{p}^{2}$ = 0.07. The error rate of the participants’ response to the in-group was significantly lower than that of the out-group. The main effect of the task was significant, *F*(1, 102) = 25.58, *p* < 0.001, ${\eta }_{p}^{2}$ = 0.20. The error rate of participants under cognitive conditions was significantly lower than that under affective conditions. The interaction was not significant.

## Discussion

Consistent with prior research findings (Rieffe et al., 2005; Shamay-Tsoory et al., 2010), our results showed that both Uygur and Han participants had a longer reaction time and higher error rate in affective ToM processing than in cognitive ToM processing, possibly because, in addition to processing the text clues and eye gaze directions, participants in the Yoni task had to also identify the target person’s facial expression, which requires individuals to call on additional cognitive resources for processing, resulting in increased reaction time and error rate.

Furthermore, Han participants showed in-group had an advantage in the reaction time of affective ToM processing, whereas Uygur participants did not. This may be because Uygurs had a deep cognitive processing effect in emotional processing, so they did not show in-group advantage in reaction time, but only show in-group advantage in error rate. Previous studies have found that although it is more difficult for individuals to identify other-race faces (Meissner & Brigham, 2001), the ability of individuals to process other-race faces can be improved with the increase in contact and familiarity with other-race faces in daily life (Mckinnon & Moscovitch, 2007; Wright et al., 2003). The proportion of the Han population is relatively large, and Uygur college students also study and communicate with many Han students at school. Therefore, the difference in the reaction time to face image processing between Uygur and Han was insignificant. However, Han students were less frequently in contact with Uygur and did not know much about their facial emotions. Therefore, Uygur people may take longer to process the affective state. Increased contact between ethnic groups may increase the accuracy of inferring the psychological state of out-group members and minimize the accuracy gap in evaluating beliefs about in-group compared to out-groups (Bjornsdottir & Rule, 2016). Accordingly, enhanced interethnic communication could improve an individual’s ability to comprehend the psychological state of different ethnic groups.

## General discussion

### The effect of ethnicity on ToM processing

Our results showed that compared to Uygur participants, Han participants had shorter reaction times and lower error rates in ToM processing. This finding aligns with prior research showing that an individual’s ToM will alter depending on their cultural background (Vu et al., 2017; Wu & Keysar, 2007). Past research has found that even in the same country, various ethnicities perform differently in ToM processing depending on their cultural background (Dong & Fu, 2007; Zheng & Ma, 2010).

Cultural differences influence various areas, including values, personality traits, visual perception, and spatial reasoning (Arnett, 2008; Henrich et al., 2010; McCrae & Terracciano, 2005). These differences may affect the development of ToM. Previous studies on Uygur personality traits indicated that Uygur students are daring, easy to take chances, eager, and forthright, which may be attributed to the nomadic lifestyle and geographical surroundings of Uygur in the past (Wang et al., 2008). Impulsiveness had a strong association with one’s ToM (Vales & Mora, 2016). Uygurs’ personality traits may influence one’s understanding and speculation of others’ psychological states, making it easier for people to make rash decisions, which could explain the differences in ToM between Uygur and Han adults. Furthermore, among the components of executive function, inhibition control had the highest correlation with the ToM (Vetter, 2013), potentially because inhibition control can compel individuals to suppress their psychological state and put themselves in another person’s psychological state. Individuals needed to use certain cognitive resources to restrain their initial egocentric state while pondering about others’ psychological states (Chen & Su, 2011). Uygur may also need to utilize the control system to solve the conflict between Uygur language and Mandarin (Wang & Zhang, 2018), who sacrificed some cognitive resources. This may also be one reason why the ToM performance of Uygur individuals was not as good as that of Han individuals.

Another reason for the difference between Uygur and Han’s participants in ToM processing was due to potential processing differences between Uygur participants and fluent Mandarin speakers. The prior research has suggested a link between ToM and linguistic aptitude (Astington & Jenkins, 1999; Shatz et al., 2003). Han participants speak Mandarin as their first language, whereas Uygurs speak it as their second. Although the task the studies implemented demanded a low language level, a processing gap between Uygur participants and fluent Mandarin speakers still existed. Uygur participants commonly used Uygur and Mandarin in their daily lives, using Mandarin and Chinese characters less frequently than Chinese participants, implying that their processing of Chinese characters is inferior to that of Han participants (Yang et al., 2019). Bradford et al. (2018) used the Self/Other Differentiation task to test Chinese and Western participants, and they did not show cross-cultural difference. What is different from our study is that they used the Chinese version for Chinese participants and English version for Western participants. Therefore, language may have an impact on Uygur’s ToM processing in our study.

### The influence of group identify on ToM processing

We found that both Uygur and Han participants could more easily infer the psychological state of their cultural group members. This finding suggested that there may be in-group advantages in cognitive ToM processing and affective ToM processing. A recent study (Gönültaş et al., 2020) investigated Turkish children’s inferring the psychological state of in-group members (Turks), similar out-group members (Syrians in Turkey), and different out-group members (Nordic). They found that individuals’ beliefs about the psychological status of in-group members were more accurate compared to the two external groups. Construal Level Theory also supported this result. When individuals represented others with different intimacy, the representation methods were different (Trop & Liberman, 2003). Others who were similar often adopted low-level explanations, paying attention to the specific and detailed characteristics of individuals or events. The intensity of their personification simulation was high so that they could infer the psychological activities of similar others more quickly (Meyer, 2013). Simultaneously, modest differences in nonverbal information expressions (Elfenbein & Ambady, 2003) and similar emotions (Baron-Cohen, 1996) among races or ethnic groups may explain this result.

## Limitations

Firstly, we did not examine the individual’s inhibitory control and personality characteristics, which may affect participants’ performance of ToM. Future research should consider the ToM in different cultural backgrounds and individual’s personality traits to further examine the reasons for the differences in the performance of the ToM. Another limitation of the current study is that the language skill of the participants was not included as a control variable. Other cognitive predictors, such as fluid intelligence, should continue to be investigated in future studies. Last but not least, ToM is a complex structure, and there may be great differences between different ToM tasks. Future studies should try to use various types of ToM tasks to be more ecologically effective.

## Conclusion

We found that task types influence Uygur and Han adults’ ToM. It was more difficult to process an individual’s ToM in the false belief condition, and it was more difficult to process ToM in an affective task compared to a cognitive task condition. Han adults performed better in cognitive ToM processing and affective ToM processing than Uygur adults, reflecting shorter reaction times and lower error rates. On the whole, Uygur and Han people have shown in-group advantage in cognitive and affective ToM. Besides, Uygur participants were more accurate in inferring the cognitive states of in-group than the out-group; Han participants inferred the emotional state of in-group faster than out-group. Our results showed that culture and group identification might affect the ToM processing. Strengthening the communication between ethnicities may enable individuals to better process out-group members’ psychological states.

## Data Availability

Not applicable.

## Appendix Questionnaire of typical names of Uygur and Han male and female students

### Male

Please choose the name you think is the most common for man of both nationalities:

Han nationalities: 王志强 (WANG zhiqiang) 张俊杰 (ZHANG junjie) 李博文 (LI bowen)

Uygur nationalities: 买买提 (Maimaiti) 阿里木 (Arimu) 尼加提 (Nigati)

### Female

Please choose the name you think is the most common for woman of both nationalities:

Han nationalities: 李文静 (LI wenjing) 陈瑞雪 (CHEN ruixue) 张佳怡 (ZHANG jiayi)

Uygur nationalities: 古丽仙 (GU lixian) 热依汗 (Reyhan) 热依曼 (Zeeman)

## Footnotes

1 "Min kao Han" means that minority students take The National College Entrance Examination in Chinese. Minority students who take the Min kao Han are mainly enrolled in general higher education schools or majors that use Chinese language and script. They have received Chinese language education since childhood, and their schooling is almost the same as that of Han Chinese students, so they can communicate, write and read in Chinese fluently that use Chinese language and script. They have received Chinese language education since childhood, and their schooling is almost the same as that of Han Chinese students, so they can communicate, write and read in Chinese fluently.
