# Correction: The influence of in-groups and out-groups on the theory-of-mind processing: evidence from different ethnic college students

**DOI:** 10.1186/s41235-023-00515-9

**Published:** 2023-09-29

**Authors:** Tingyu Zhu, Lijin Zhang, Ping Wang, Meiqiu Xiang, Xiujuan Wu

**Affiliations:** 1 School of Psychology, Shaanxi Normal University, No. 199, Chang’an South Road, Yanta District, Xi’an, 710062 Shaanxi Province China; 2 Shaanxi Provincial Key Research Center of Child Mental and Behavioral Health, Xi’an, China; 3 Shaanxi Key Laboratory of Behavior and Cognitive Neuroscience, Xi’an, China; 4 School of Educational Science, Liupanshui Normal University, Liupanshui, China

**Correction to: Cognitive Research: Principles and Implications (2023) 8:5** **https://doi.org/10.1186/s41235-023-00461-6**

The original article (Zhu et al., 2023) mistakenly omitted Funding information. The corrected funding information is presented ahead:
